# Optimization of personalized therapies for anticancer treatment

**DOI:** 10.1186/1752-0509-7-31

**Published:** 2013-04-12

**Authors:** Alexei Vazquez

**Affiliations:** 1 Department of Radiation Oncology and Center for Systems Biology, The Cancer Institute of New Jersey, University of Medicine and Dentistry of New Jersey, Robert Wood Johnson Medical School, 195 Little Albany St, New Brunswick, NJ 08903, USA

**Keywords:** Cancer, Personalized medicine, Targeted therapy, Combinatorial therapy

## Abstract

**Background:**

As today, there are hundreds of targeted therapies for the treatment of cancer, many of which have companion biomarkers that are in use to inform treatment decisions. If we would consider this whole arsenal of targeted therapies as a treatment option for every patient, very soon we will reach a scenario where each patient is positive for several markers suggesting their treatment with several targeted therapies. Given the documented side effects of anticancer drugs, it is clear that such a strategy is unfeasible.

**Results:**

Here, we propose a strategy that optimizes the design of combinatorial therapies to achieve the best response rates with the minimal toxicity. In this methodology markers are assigned to drugs such that we achieve a high overall response rate while using personalized combinations of minimal size. We tested this methodology in an *in silico* cancer patient cohort, constructed from in vitro data for 714 cell lines and 138 drugs reported by the Sanger Institute. Our analysis indicates that, even in the context of personalized medicine, combinations of three or more drugs are required to achieve high response rates. Furthermore, patient-to-patient variations in pharmacokinetics have a significant impact in the overall response rate. A 10 fold increase in the pharmacokinetics variations resulted in a significant drop the overall response rate.

**Conclusions:**

The design of optimal combinatorial therapy for anticancer treatment requires a transition from the one-drug/one-biomarker approach to global strategies that simultaneously assign makers to a catalog of drugs. The methodology reported here provides a framework to achieve this transition.

## Background

*Personalized cancer therapy* has been proposed as the next battle in the war on cancer and targeted therapies as the new warfare machinery [1]. *Targeted therapies* are designed to treat cancers carrying specific molecular alterations. In turn these molecular alterations can be used as *companion biomarkers* to inform the decision of using, or not using, the targeted therapy to treat a patient [2]. For example, in the context of breast cancer, the level of the receptor tyrosine kinase HER2/*neu* is used to select trastuzumab (Herceptin; Genentech) as adjuvant therapy [3].

By design, a targeted therapy is expected to be effective in a subset of cancer patients (e.g., trastuzumab in HER2/*neu* positive breast cancer patients). However, even within this subset, the long-term response may be reduced. Some patients may initially respond to the targeted therapy but later on regress due to the occurrence of secondary molecular alterations. For example, in the context of melanoma, cancers with the *BRAF*(*V600E*) mutation can be treated with vemurafenib (Zelboraf, Plexxikon) resulting in outstanding response [4]. However, in about one year most patients regress due to upregulation of compensatory pathways [5,6]. The molecular background of a cancer can also modulate the response to a targeted therapy, even when treatment is suggested by the biomarker. For example, as a difference with melanoma patients, colon cancer patients harbouring the same *BRAF*(*V600E*) mutation show a very limited response to vemurafenib [7]. One mechanism explaining this difference is the feedback activation of EGFR upon treatment with vemurafenib and the fact that EGFR levels are higher in colon cancer than in melamoma cells [8].

Although targeted therapies may fail as single agents, they can still be effective when used in combination with other agents. *Combinatorial therapy* is a rational approach to overcome the failure of single drugs [9]. One hypothesis is that one agent in the combination can cover for the caveats of other agents, increasing the response rate [10]. As for the case of single agents, biomarkers can be used to inform the inclusion of targeted therapies in a drug combination, which we name *personalized combinatorial therapy*.

The shift from single drug targeted therapy to combinatorial personalized therapies introduces a new challenge. As today, there are hundreds of targeted therapies with their associated biomarkers, some of which are already in use to inform treatment decisions. If we would consider the whole arsenal of targeted therapies as a treatment option for every patient, very soon we will reach a scenario where each patient is positive for several markers suggesting their treatment with several targeted therapies [11]. Given the documented side effects of anticancer drugs, it is clear that such a strategy is unfeasible. A new strategy is needed to optimize the design of combinatorial therapies to achieve the best respond rates with the minimal toxicity. In this work we introduce a methodology to achieve this goal.

## Results and discussion

**Figure 1:**
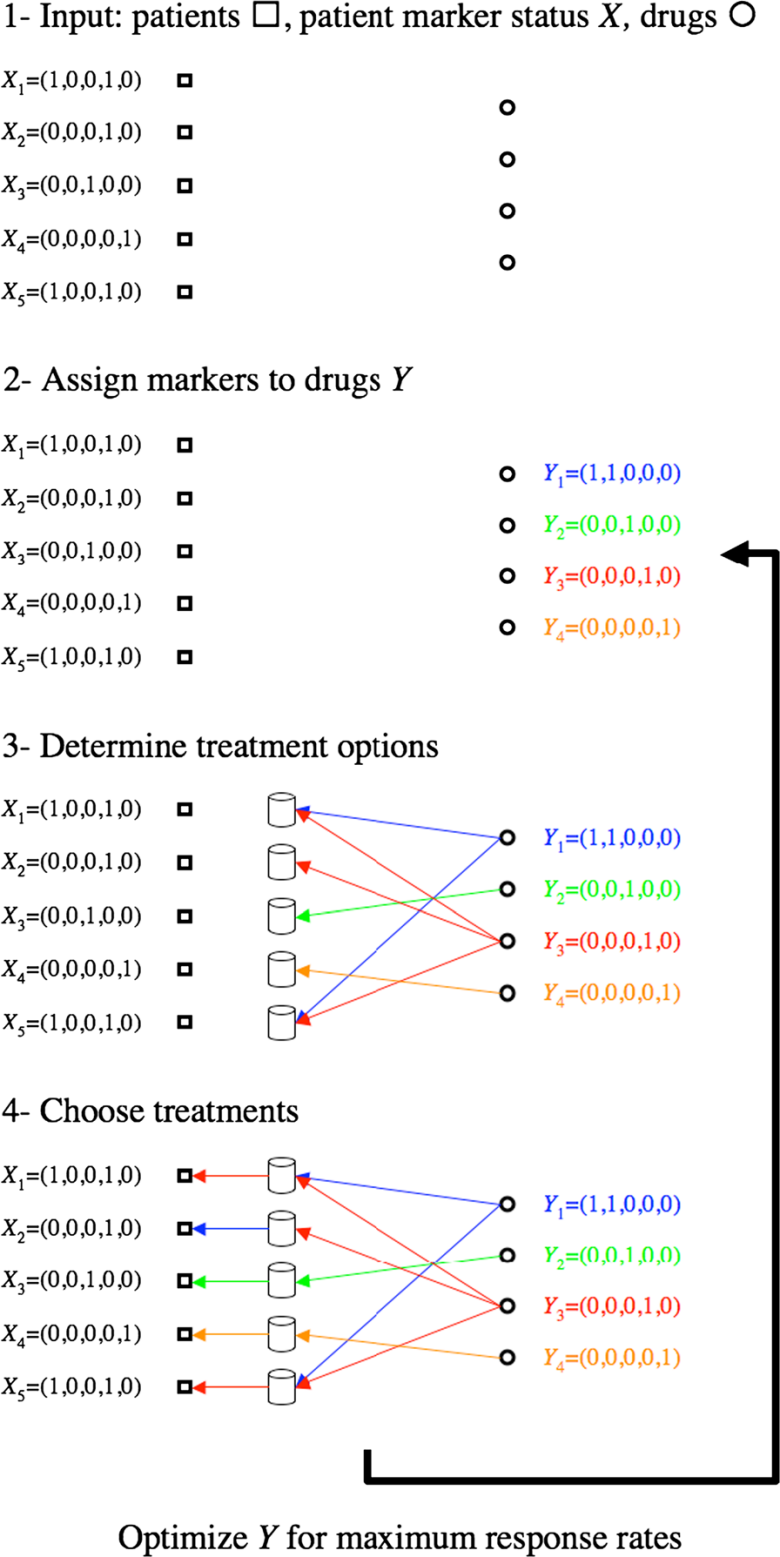
**Optimization of personalized therapies.** 1- We are given as input a set of patients, Boolean vectors reporting the markers status on each patient (*X*) and a set of drugs available for treatment. 2- A Boolean vector reporting the markers that will be used to inform treatment is specified for each drug (*Y*). 3- Drugs are suggested for the treatment of each patient using a drug-to-sample protocol depending on the sample and drug markers (*f*_*j*_(*X*_*i*_,*Y*_*j*_)). In this example a drug is suggested for the treatment of a patient whenever they share at least one marker. 4- Finally, a sample protocol is used to specify the treatment to each patient (*g*). In this example the best treatment for each sample is selected. Finally, we optimize the marker assignments to drugs (*Y*_*j*_), the drug-to-sample protocols (*f*_*j*_(*X*_*i*_,*Y*_*j*_)) and the sample protocol (*g*) to obtain the maximum overall response rate, as represented in the figure by the arrow.

The shift from single drug targeted therapy to personalized combinatorial therapies introduces a new challenge. We need to define a protocol to design the personalized combinations given a catalog of drugs, a catalog of markers and the status of those markers in the patient’s cancer. To formally address this problem we introduce the scheme depicted in Figure 1. We are given as input a cohort of patients together with the status of *m* markers in those patients. To be more precise, the markers status of each patient is represented by a barcode or Boolean vector *X*_*i*_=(*x*_*i*1_,…,*x*_*im*_), where *x*_*il*_=1 when marker *l* is observed in patient *i* and 0 otherwise. We are also given as input a set of drugs that are available for anticancer treatment. In the context of personalized medicine we would like to assign markers to a drug to identify the patient subpopulation with the best response rates. Again, to be precise, the marker assignment to each drug is represented by a barcode or Boolean vector *Y*_*j*_=(*y*_*j*1_,…,*y*_*jm*_), where *y*_*jl*_=1 if marker *l* is used to inform the treatment with drug *j* and 0 otherwise. A drug-to-sample protocol *f*_*j*_(*X*_*i*_,*Y*_*j*_) is used to inform the treatment options, where *f*_*j*_(*X*_*i*_,*Y*_*j*_)=1 indicates to consider drug *j* as a treatment option for sample *i* and *f*_*j*_(*X*_*i*_,*Y*_*j*_)=0 otherwise. For example, Figure 1 illustrates the protocol where *f*_*j*_(*X*_*i*_,*Y*_*j*_)=1 if the sample and the drug share a marker in common. Once the treatment options are determined for each sample, we then apply a patient protocol *g* to choose the personalized therapies for each patient. For example, Figure 1 illustrates the protocol *g* indicating the treatment with the drug with highest expected response rate among the treatment options identified for each patient (*g*_*best*,1_). Another possibility is to treat with the *c* drugs with the higher response rates among those suggested for each patient (*g*_*best*,*c*_).

The current approach to targeted therapies is to assign markers to drugs based either on the target for which the drug was developed or some preliminary study suggesting an increase response rate in patients having the marker. We take a more general approach where the markers are assigned to drugs to maximize the response rate to therapy. To this end, we define the following optimization problem:

Find the drug marker assignments *Y*_*j*_, the drug-to-sample protocols *f*_*j *_and sample protocol *g* that maximize the overall response rate *O*.

### Response model

To calculate *O* we require the probability that each patient responds to a drug when the drug is used as a single agent and some quantification of drug interactions. In the simplest scenario where there are no drug interactions, the probability *P*_*i*_ that a patient responds to is personalized therapy is given by the probability that it responds to at least one of the drugs on its personalized combination

(1) $$P_{i}=1-\prod_{j=1}^{d}{\left(1-p_{\text{ij}}\right)}^{e_{\text{ij}}}$$

where *e*_*ij*_=1 if drug *j* is included in the personalized therapy of patient *i* and *p*_*ij *_is the probability that patient *i*responds to drug *j* when the latter is used as a single agent. When interactions are present we can improve on (Eq. 1) after adding correction terms accounting for two-drug interactions and so on

(2) $$P_{i}=1-e^{\sum_{j=1}^{d}e_{\text{ij}}\ln\left(1-p_{\text{ij}}\right)+\sum_{j=1}^{d-1}\sum_{k=j+1}^{d}e_{\text{ij}}e_{\text{ik}}J_{\text{jk}}+\cdots }$$

In this equation values of *J*_*jk*_<0 will result in response rates higher than what expected if the drugs do not interact (synergy) while values of *J*_*jk*_>0 will result in response rates lower than what expected if the drugs do not interact (antagonism). We note that antagonism could take place at the level of pharmacodynamics (antagonism at the cellular level) or at the level of pharmacokinetics (antagonism at the drug metabolism level) and the latter may result in increased toxicity.

The average of *P*_*i *_across samples defines the overall response rate *O* of the personalized combinatorial therapies

(3) $$O=\frac{1}{s}\sum_{i=1}^{s}P_{i}$$

We are aware of documented examples of drug interactions in the context of cancer treatment [12]. However, for most combinations we do not have a quantitative estimate of how these interactions affect the response rate. For the purpose of illustrating our methodology, we will use the non-interacting drugs approximation (Eq. 1) in our simulations.

### Response-by-marker approximation

In the clinical practice we cannot test the response of each cancer patient to each approved anticancer drug. However, we can estimate the response rate to a drug depending on the present/absence of the markers assigned to that drug. For example, let us consider the case where *K*_*j *_markers are used to inform the treatment with drug *j*. The patients are divided into $2^{K_{j}}$ groups depending on the status of those markers. We can conduct a clinical trial to estimate the response rate *q*(*j*,*s*) of drug *j* for each group of patients. Once the *q*(*j*,*s*) are known, we can estimate the response rate to any patient. To be more precise we enumerate the patient groups using the index

(4) $$s_{j}\left(x\right)=\sum_{k=1}^{K_{j}}x_{l_{k}}2^{k-1}$$

where $\left(l_{j_{1}},\ldots ,l_{j_{K_{j}}}\right)$ is the list of markers assigned to drug *j* and *x*_*l *_is the status of the *l*-th marker. Using this notation we obtain the response by-marker approximation

(5) $${\bar{p}}_{\text{ij}}=q\left(j,s_{j}\left(X_{i}\right)\right)$$

In short, the probability that a given patient *i* responds to a given drug *j* is approximated by the estimated fraction of patients that responds to that drug within the group of patients having the same status as patient *i* for the markers assigned to drug *j*.

### Finding the optimal personalized combinations

We need some procedure to find the optimal treatment combinations. In the Methods section we report a simulated annealing algorithm that performs an exploration of the space of markers assigned to drugs and drug-to-sample protocols with a gradual increased bias towards improvements on the overall response rate. Although this algorithm may not find the optimal solution, it can provide a good approximation to hard computational problems [13].

### Updating the drug-to-sample protocols

During the optimization procedure we need to explore different marker assignments to drugs and different choices of drug-to-sample protocols. To this end we need some precise representation of the Boolean functions and the transformations among them. The drug-to-sample protocols are represented by a Boolean function *f*_*j*_(*X*_*i*_,*Y*_*j*_) that returns 0 (do not suggest) or 1 (suggest) depending on the status of the markers assigned to the drug on a given sample. For computational convenience it is easier to write the Boolean functions as $f_{j}\left(X_{i},Y_{j}\right)=f_{j}\left(X_{il_{1}},\ldots ,X_{il_{K_{j}}}\right)$, where *K*_*j*_ is the number of markers assigned to drug *j*, $l_{j1},\ldots l_{K_{j}}$ is the list of markers assigned to drug *j* and *f*_*j*_ is a Boolean function of *K*_*j *_inputs. Given *K* markers there are 2^k^ possible input states (*x*_1_,…,*x*_k_), which can be enumerated as follows: $a\left(x\right)=\sum_{k=1}^{K}x_{k}2^{k-1}$. For each of these input states we can set the output *o*_*a *_to 0 or 1. We can enumerate the Boolean functions with *K* inputs using the mapping $b\left(o\right)=\sum_{a=0}^{2^{K}-1}o_{a}2^{a-1}$. Therefore, we can represent every Boolean function with two indexes (*K*,*b*), the first one denoting the number of inputs and the second one the specific Boolean function with *K*-inputs.

**Figure 2:**
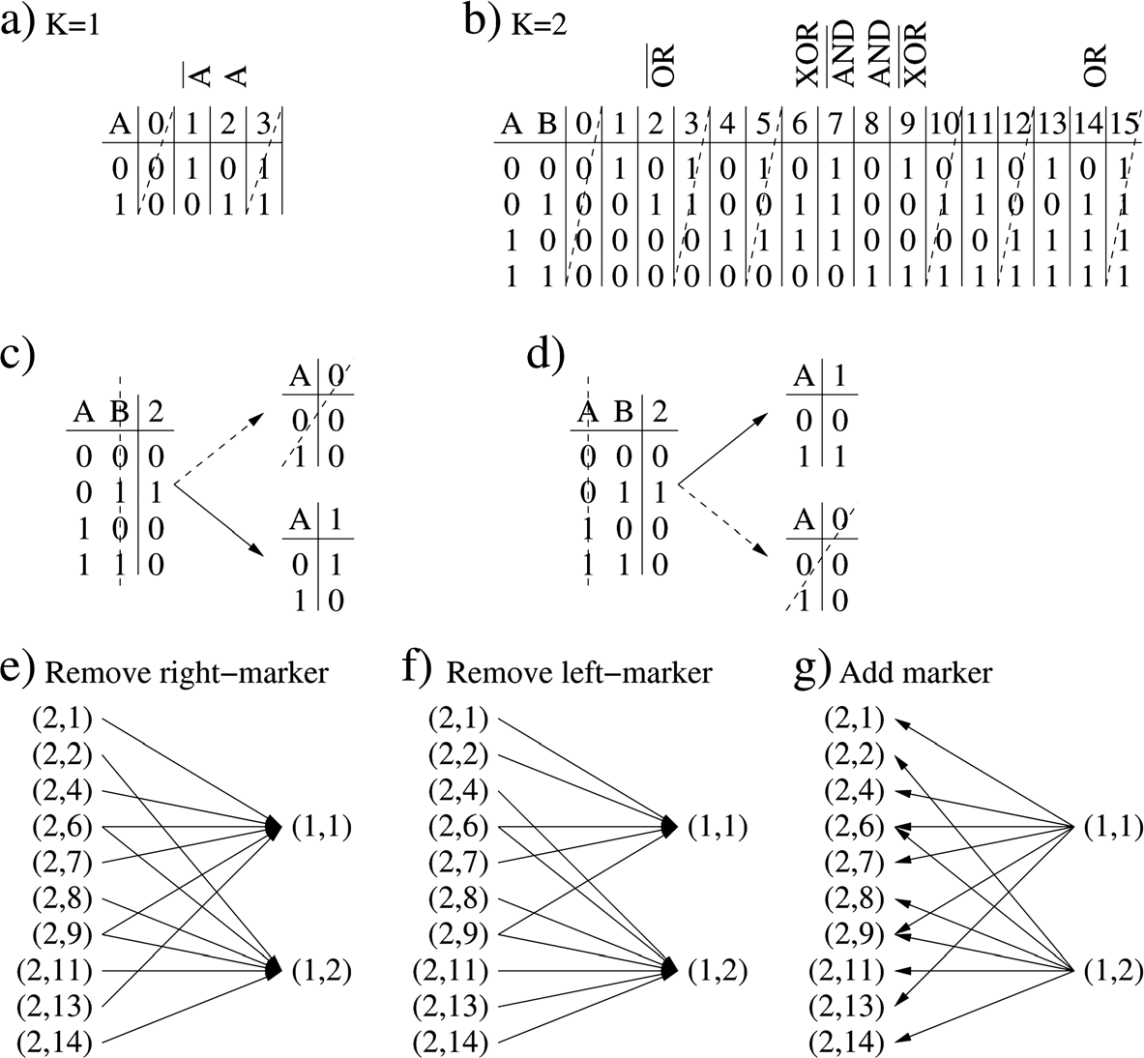
**Boolean functions and operations among them. a**) Boolean functions with one input. Functions with a dashed line are not considered because the output is independent of the input. **b**) Boolean functions with two inputs. Functions with a dashed line are not considered because the output is independent of at least one input. **c**) An example showing the removal of the right marker (B) from function (2,2), which can be either result into function (1,0) or (1,1). Since function (1,0) is excluded then the function (1,1) is always chosen. **d**) Same example but removing the left marker (A). **e**) All mappings from (2,*b*) to (1,*b*’) following removal of the right (B) marker. f) All mappings from (2,*b*) to (1,*b*’) following removal of the left marker. e) All mappings from (1,*b*) to (2,*b*’) following the addition of a marker. For simplicity, we have chosen the reverse of the right-marker removal (panel e) as the mapping for the marker addition.

Figure 2a and b show all Boolean functions with one (1,*b*) and two (2,*b*) inputs, respectively. Each Boolean function is represented by a truth-table where for each imput the output 0 or 1 is specified. The letters A and B are used to denote the inputs and the *b* index of each function is indicated on the upper raw of the truth-table. We note that functions where the output is independent of at least one input are not considered, because they can be reduced to a simpler function. For example function (1,0) (Figure 2a) is equivalent to have no markers assigned and function (2,3) (Figure 2b) is equivalent to (1,1) (Figure 2a) after removing the marker B.

To explore different Boolean functions we change the function, add a new marker or remove one marker. When changing a Boolean function, (*K*,*b*)→(*K*,*b*’), a new function is selected at random among all considered Boolean functions with the same number of inputs. When removing a marker, (*K*,*b*)→(*K*-1,*b*’), if the drug has one marker then we remove it, the drug will have no markers assigned and, therefore, it will not be considered for the treatment of any patient. If the drug has two markers assigned then we remove one of the two markers and use the transformations illustrated in Figure 2c and d. For example, in Figure 2c we start with the function (2,2) and remove the B (right) input. For this function the output is always 0 when the A (left) input is 1 but the output can be 0 or 1 when the A input is 0. Therefore, (2,2) can be mapped to (1,0) or (1,1) after removing the B input. Since the output of (1,0) is independent of the input state it is not considered. A similar reasoning can be applied to obtain the mappings for function (2,2) when removing the A marker instead (Figure 2d). Applying this approach to every (2,*b*) function we obtain the mappings in Figure 2e and f. Finally, if a marker is added, (*K*,*b*)→(*K*+1,*b*’), then we use the mappings in Figure 2g, which are the reverse of (*K*-1,*b*’)→(*K*,*b*) removing the A (left) input. In all cases, when more that one choice is available we choose one of them with equal probability.

### Case study

**Figure 3:**
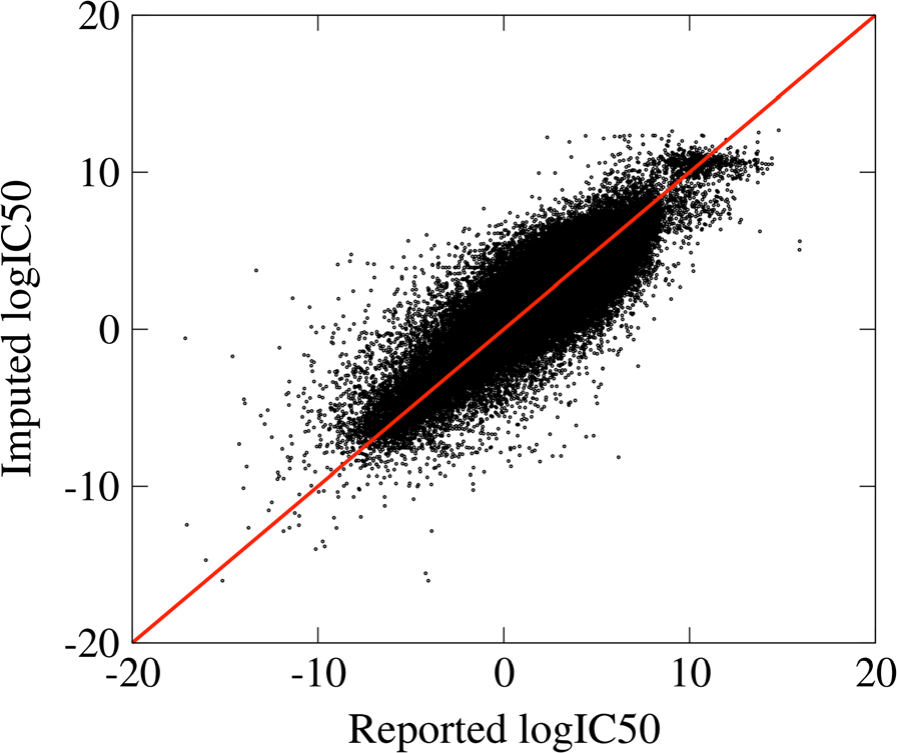
**Comparison between the imputed and reported logIC50 data.** The solid line represents the case when the imputed and reported values coincide.

To test our methodology we investigate an *in silico* case study where we can actually quantify the response of each sample to each drug. The *in silico* case study is based on in vitro growth inhibition data reported by the Sanger Institute [14]. In the Sanger screen 714 cell lines were tested for their responses against 138 drugs. For several sample-drug pairs the natural logarithm of the drug concentration to achieve a 50% growth inhibition relative to untreated controls (logIC50) was reported. The logIC50 data is missing for 26,031 drug-cell line pairs, representing 20% of all drug-sample pairs. The missing logIC50 data was imputed using the weighted average approach described in the Methods section. The Pearson Correlation Coefficient (PCC) between the imputed and actual log50s, when the latter were available, was 0.89 (Figure 3).

For each cell line the cancer subtype and the status of 47 cancer related genes was also reported, including somatic mutations and copy number alterations. We use as markers the observation of a specific cancer type (e.g., breast cancer), somatic mutations (e.g., *TP53*:*wild*-*type*, *TP53*:*R175H*, *TP53*:*R248Q*, etc.), and copy number alterations (*gene*:-, *gene*:*0*, *gene*:+ for deletion, normal and amplification, respectively). This procedure resulted in 921 markers. Among those, we retained 181 markers that are observed in at least 10 cell lines.

To each cell line we associate a sample that is fully composed of that cell line. We assume that different drugs are used at different treatment doses because they are active at different concentration ranges. The mean logIC50 of a drug across cancer cell lines is a good estimate of the typical concentration for the drug activity in this *in vitro* setting. Thus, for each drug we set the treatment log-concentration *y*_*j*_=mean(logIC50)_*j*_+log*h*, where *h* represents the fold change in the dose. Values of *h* below 1 represent low dose therapy, while those above 1 represent high dose therapy. In average, cancer cells have IC50s that are about 2 fold lower than those of normal cells [15]. Based on this we assume that the highest tolerated dose is *h*=2, and that is the dose used for treatment.

We assume that due to variations in drug delivery the actual log-dose reaching the cancer cells, denoted by *Z*_*j*_, is different from *y*_*j*_. Pharmacokinetic variables generally follow a normal distribution after a log-transformation [16] and, therefore, we assume that *Z*_*j *_(the log-dose) is a random variable following a normal distribution, with mean *y*_*j *_and variance σ. Here σ models variations associated with drug pharmacokinetics in patients. Pharmacokinetic parameters characterizing the steady state plasma drug concentrations and drug clearance rates can vary as much as 2–10 fold [17,18]. To model such variations we will use σ=1,10.

We define a response as the achievement of at least 50% growth inhibition. In this case a sample responds to a drug if *Z*_*j*_>logIC50_*ij *_and does not respond otherwise. Under these assumptions, the probability *p*_*ij *_that sample *i* responds to drug *j* is given by

(6) $$p_{\text{ij}}=\frac{1}{2}\text{erfc}\left(\frac{\log\mathrm{IC}50_{\text{ij}}-\text{mean}{\left(\log\mathrm{IC}50\right)}_{j}-\log h}{\sqrt{2}\sigma }\right)$$

where erfc(*x*) is the complementary error function. When the cell line logIC50_*ij*_ is much higher than the treatment dose reaching the cancer cells (logIC50_*ij*_-*y*_*j*_>>σ) then *p*_*ij*_≈0. In contrast, when the cell line logIC50_*ij *_is much lower than the treatment dose reaching the cancer cells (logIC50_*ij*_-*y*_*j*_<<σ) then *p*_*ij*_≈1.

To test a more realistic scenario, we are not going to use the response probabilities in (Eq. 6). Instead, we are going to use the response by-marker approximation in (Eq. 5). To this end, given a drug and its assigned markers, we divide the cell lines into groups depending on the status of those markers, and estimate the response probability of *q*(*j*,*s*) as the average of *p*_*ij *_over all cell lines in that group. To avoid biases from small group sizes, we set *q*(*j*,*s*)=0 for any group with less than 10 samples.

We do not have an estimate of the possible interactions between the 138 drugs in this *in silico* study. We assume that the drugs do not interact and we approximate the response to a personalized drug combination by (Eq. 1), but replacing *p*_*ij*_ by the response by-marker approximation (Eq. 5).

In the optimization problem defined above we could attempt to optimize the marker assignments to drugs, the drug-to-sample protocols *f*_*j*_(*X*_*i*_,*Y*_*j*_) and the sample protocol *g*. However, to reduce the computational complexity of the problem, we will impose the sample protocol *g*_*best*,*c*_, assign at most two markers to each drug and optimize over marker assignments to drugs and the drug-to-sample protocols.

**Figure 4:**
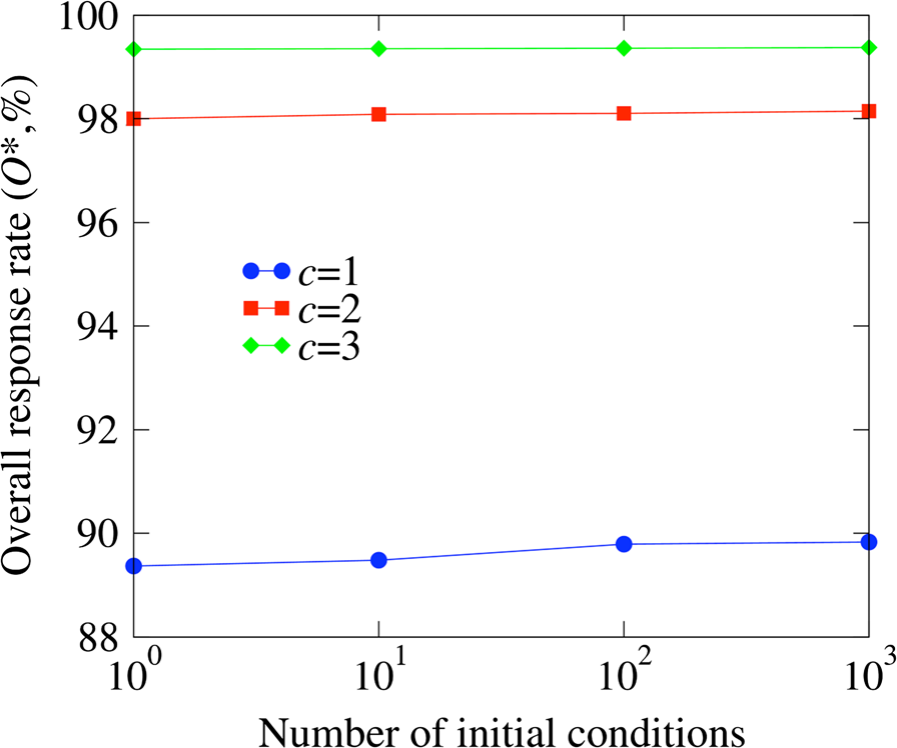
**Convergence of the simulated**-**annealing algorithm for the *in silico *study.** The overall response rate (as estimated with the by-marker approximation, O*) as a function of the number of initial conditions tried.

Using the simulated-annealing algorithm we obtained the optimal personalized therapies for the *in silico* cohort. In general we have no way to warranty that the simulated-annealing algorithm did not get stuck at a local minimum, precluding it from finding the optimal solution. However, by starting at different initial assignments of markers/Boolean-functions and monitoring the improvement on the solutions found we can get an idea of how close we are from the optimal solution. Figure 4 shows the highest overall response rate (as estimated with the by-marker approximation, O*) as more initial conditions were tested. There are no significant improvements between a 100 and 1,000 initial conditions indicating that the simulating-annealing algorithm is close to the optimal solution.

We note that in this study we count with the actual response probability of each cell line to each drug. Therefore, we can use as input the optimal personalized combinations obtained by using the response by-marker approximation (Eq. 5) and then calculate the overall response rate using the original cell line response rates (Eq. 6).

**Figure 5:**
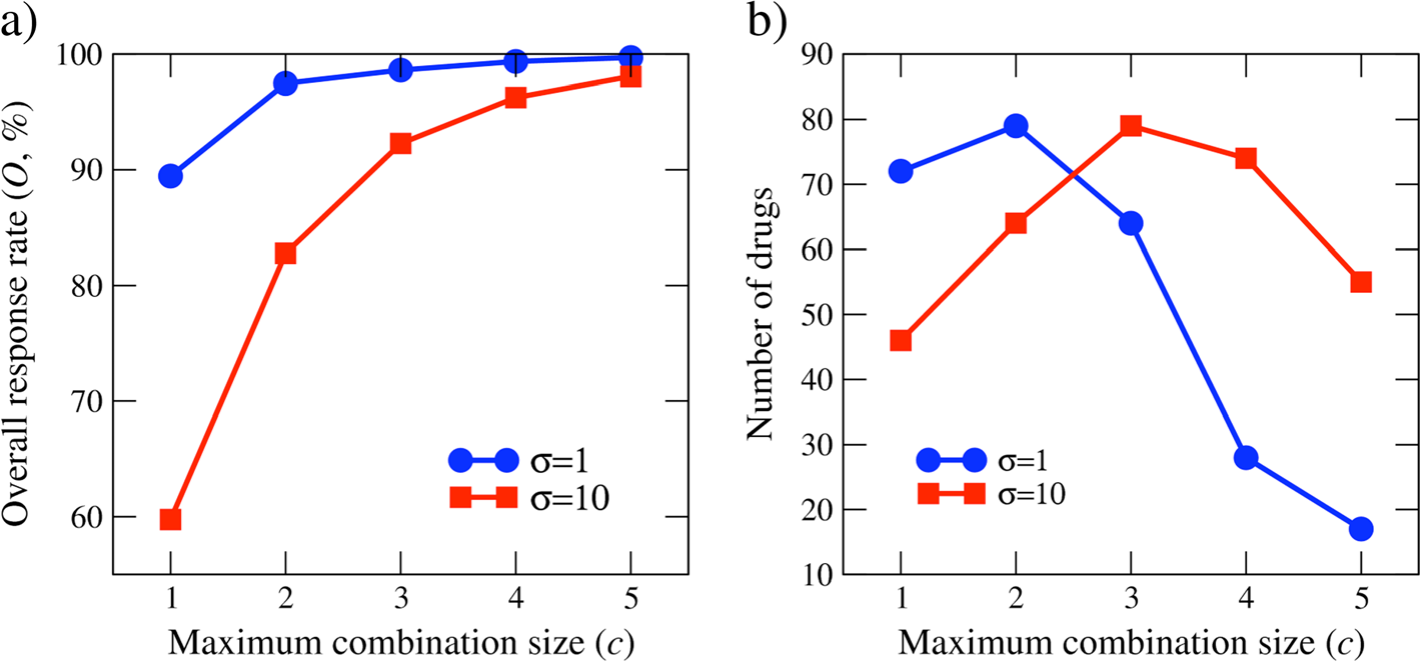
**Predictions of the *in silico study*.** Model predictions as a function of the maximum combination size allowed for two values of the pharmacokinetic variations parameter σ. **a**) The overall response rate. **b**) Number of drugs used for the treatment of at least one sample.

When the pharmacokinetic variations are small (σ=1), the predicted overall response rate is as high as 90% when treating with personalized therapies using one drug alone. Then it increases towards 100% as we move to personalized combinations using more drugs (Figure 5a). However, a 10-fold increase in the pharmacokinetic variations (σ=10) results in a drop of the overall response rate to about 60% when treating with one drug alone (Figure 5a). This observation indicates that the success of personalized therapy will also depend on the magnitude of pharmacokinetic variations and on our ability to personalize the drug dosage for each patient to counteract those pharmacokinetic variations.

We note that not all drugs are included in the treatment of at least one sample, resulting in a smaller effective drug catalog (Figure 5b). For all the maximum combination sizes tested, less than 80 out of 138 (58%) of the drugs are needed. Furthermore, beyond personalized combinations of three drugs, we observe a decrease in the number of needed drugs as we increased the maximum allowed combination size (Figure 5b). This observation suggests that the need for only 58% of the drugs will hold for larger combination sizes. We note that the decrease of the needed drugs is unexpected. For example, if the response rates were independent identically distributed random variables then the probability that a drug is selected for the treatment of a samples is *c*/*d*, the probability that a drug is selected for the treatment of at least one sample is 1-(1-c/d)^s^ and the average number of drugs used for the treatment of at least one sample is d* = *d*[1 − (1 − *c*/*d*)^*s*^]. Therefore, for independent identically distributed response rates d* increases monotonically with increased the combination size *c*. The departure from this expectation in Figure 5b could be due to the existence of correlations in the response rates of different drugs when treating different cells lines. Furthermore, we cannot exclude that for large *c* the simulated-annealing algorithm gets trapped in local optima and that for the actual global optimal d* does increases with increasing *c*. In any event this discrepancy should motivate future work to obtain theoretical estimates for d* based on the patterns of correlations between the response rates and the ability of the simulating-annealing algorithm to reach the global optimum.

In Table 1 we report the effective drug catalog for the small pharmacokinetic variations case (σ=1) and maximum combination size *c*=3 drugs. In addition, we report whether those drugs were included in the catalogs for *c*=1 and 2, showing the percent of samples treated when included and (−) otherwise. Most drugs in the *c*=3 catalog are also included in the *c*=1 and 2 catalogs, indicating that there is a core set of drugs that is relevant independent of the maximum combination size allowed. The percentage of samples treated with a given drug in the catalog increases from *c*=1 to 3. This effect can be explained by the fact that, as we allow combinations of more drugs, a drug can be included in personalized combinations as a second or third choice.

**Table 1:** The catalog of drugs in the optimized personalized combinatorial therapies

	**% of samples treated**			***K***	***f***	**Markers**	**Target**
***c***	**1**	**2**	**3**	**3**	**3**	**3**	**3**
Embelin	0.7	5.9	31.5	2	13	lung: small_cell_carcinoma,TP53:wt	XIAP
Nutlin-3a	4.1	8.0	24.5	2	11	TP53:wt,RB1:wt	MDM2
Bicalutamide	1.3	3.9	21.4	2	11	ALK:wt,KRAS:0	Androgen receptor (ANDR)
XMD8-85	1.0	5.7	19.5	2	9	CDKN2A:wt,malignant_melanoma	ERK5 (MK07)
Shikonin	1.7	1.7	11.8	1	2	TP53:wt	unknown
NVP-BEZ235	0.6	-	8.0	2	4	PTEN:wt,EZH2:wt	PI3K (Class 1) and mTORC1/2
CI-1040	1.8	3.8	7.6	2	6	malignant_melanoma,TP53:p.R273H	MEK1/2
EHT 1864	1.1	3.5	7.1	1	2	lung: small_cell_carcinoma	Rac GTPases
BMS-754807	-	4.1	7.0	2	14	neuroblastoma,KRAS:p.G12V	IGF1R
PLX4720	0.8	3.5	6.9	2	13	BRAF:p.V600E,MSH2:wt	BRAF
BX-795	0.7	5.7	6.3	2	14	glioma,KRAS:+	TBK1, PDK1, IKK, AURKB/C
AKT inhibitor VIII	2.4	5.6	6.3	2	9	lung: NSCLC: adenocarcinoma,EGFR:wt	AKT1/2
AZD6482	3.8	6.2	6.3	1	2	glioma	PI3Kb (P3C2B)
RDEA119	3.9	7.4	6.0	2	13	malignant_melanoma,BRCA1:0	MEK1/2
MS-275	4.9	5.9	5.9	2	6	lung: small_cell_carcinoma,RB1:wt	HDAC
BI-D1870	0.7	1.7	5.3	2	14	CCND1:0,MYCN:0	RSK1/2/3/5, PLK1, AURKB
MG-132	1.4	-	5.2	1	2	glioma	Proteasome
FH535	1.5	3.6	5.0	2	14	breast,CCND1:+	unknown
Docetaxel	2.8	1.3	4.6	2	9	upper_aerodigestive_tract,EGFR:wt	Microtubules
CGP-60474	-	2.8	4.3	1	2	CDKN2a(p14):p.?	CDK1/2/5/7/9
AS601245	1.7	2.5	4.2	2	7	ovary,osteosarcoma	JNK
NVP-TAE684	1.5	3.8	4.2	1	1	APC:wt,stomach	ALK
Epothilone B	1.3	1.4	4.1	2	7	PIK3CA:p.E545K,TP53:p.R248W	Microtubules
Camptothecin	2.9	3.8	4.1	2	7	AML,lymphoblastic T cell leukaemia	TOP1
Vorinostat	4.1	5.7	4.1	1	2	MYCN:+	HDAC inhibitor Class I, IIa, IIb, IV
A-443654	1.4	3.6	4.1	1	1	SMAD4:wt	AKT1/2/3
PD-0325901	1.1	2.1	3.9	2	11	large_intestine,VHL:0	MEK1/2
RO-3306	1.5	3.2	3.9	2	11	cervix,MYCL1:0	CDK1
17-AAG	2.2	2.2	3.8	2	6	STK11:wt,MET:0	HSP90
S-Trityl-L-cysteine	1.1	3.5	3.8	1	1	FBXW7:wt	KIF11
ZM-447439	0.1	1.3	3.6	2	7	lung: NSCLC: large cell,RB1:-	AURKB
Vinblastine	1.4	2.2	3.2	2	13	upper_aerodigestive_tract,IDH1:0	Microtubules
Paclitaxel	0.1	2.5	3.2	2	11	oesophagus,TSC1:wt	Microtubules
AICAR	0.6	1.7	2.9	1	1	KDM6A:wt	AMPK agonist
BIBW2992	1.4	2.2	2.9	1	1	ERBB2:0	EGFR, ERBB2
JNK-9L	-	-	2.7	1	2	AML	JNK
BAY 61-3606	1.0	2.1	2.7	1	2	Ewings sarcoma	SYK
AMG-706	-	-	2.7	1	2	Ewings sarcoma	VEGFR, RET, c-KIT, PDGFR
AZ628	-	2.2	2.5	2	9	KRAS:p.G12D,FGFR3:0	BRAF
BMS-536924	-	1.1	2.5	2	7	KRAS:+,MDM2:+	IGF1R
JW-7-52-1	1.1	1.7	2.5	1	2	stomach	MTOR
Elesclomol	1.0	3.5	2.4	2	8	bladder,TSC1:wt	HSP70
Pyrimethamine	0.8	3.5	2.4	1	2	pancreas	Dihydrofolate reductase (DHFR)
KIN001-135	0.3	0.7	2.2	1	1	MET:+	IKKE
Dasatinib	-	-	2.0	2	13	Renal cell carcinoma,NRAS:0	ABL, SRC, KIT, PDGFR
ABT-888	1.5	2.5	1.7	2	11	lymphoid_neoplasm other,CDK4:0	PARP1/2
BI-2536	2.9	3.1	1.7	2	2	CDKN2A:p.0?,MYC:0	PLK1/2/3
IPA-3	-	-	1.7	1	2	B cell lymphoma	PAK
WO2009093972	1.5	2.0	1.7	1	2	soft tissue other	PI3Kb
Methotrexate	1.7	4.2	1.5	2	8	lymphoblastic leukemia,GNAS:wt	Dihydrofolate reductase (DHFR)
Roscovitine	0.1	3.1	1.5	1	2	Burkitt lymphoma	CDKs
FTI-277	1.4	1.4	1.5	1	2	thyroid	Farnesyl transferase (FNTA)
PAC-1	1.5	1.5	1.5	1	2	Burkitt lymphoma	CASP3 activator
CCT018159	1.3	3.5	1.4	2	14	osteosarcoma,PTEN:0	HSP90
PF-4708671	0.7	0.8	1.4	2	13	Myeloma,BRCA1:wt	p70 S6KA
TW 37	0.8	1.1	1.4	2	6	MLH1:wt,APC:0	BCL-2, BCL-XL
MK-2206	1.3	1.4	1.4	1	2	endometrium	AKT1/2
JNK Inhibitor VIII	-	-	0.3	1	2	AML	JNK
Obatoclax Mesylate	-	-	0.1	2	2	RB1:-,CDK6:+	BCL-2, BCL-XL, MCL-1

Drugs used for the treatment of at least one sample for maximum combination size *c*=3 and pharmacokinetic variations parameter σ=1. For each drug we report the drug-name and the percentage of samples treated with that drug (%). In the cases where the drugs were also included in the *c*=2 and 3 catalogs, the percentage of samples treated are reported as well. For the maximum combination size *c*=3 we also report the number of assigned markers *K*_*j*_, the assigned drug-to-sample protocol *f*_*j*_, the assigned markers, and the drug target.

We note that in some instances the marker assigned to a drug coincides with what expected given the known drug target (Table 1, Markers and Target columns). For example, the marker TP53:wt (i.e., TP53 wild-type) is suggested to inform the treatment with nutlin-3a. This makes sense because nutlin-3a releases TP53 from the inhibition by its negative regulator MDM2 and the outcome of nutlin-3a treatment is modulated by the TP53 status [19]. In another case, the marker BRAF:V600E is assigned to the BRAF inhibitor PLX4720 [20]. The marker KRAS:G12D is assigned to another BRAF inhibitor, AZ628, which still makes sense because KRAS is just upstream of BRAF in the RAS/RAF/MAPK/ERK signaling pathway [21]. In another case, the marker ERBB2:0 (i.e., normal ERBB2 copy number) and the Boolean function (1,1) (i.e., suggest in the absence of the marker) are assigned to the ERBB2/EGFR inhibitor BIBW2992, which again makes sense since ERBB2 inhibitors are expected to be more effective in the presence of ERBB2 amplifications [22]. However, in most instances the relation between the assigned marker/Boolean-function and the known target is not obvious. The best example is the assignment of a tissue type as a marker, rather than the status of the gene coding for the target or another gene in the same pathway.

## Conclusions

We have proposed a methodology that optimizes the assignment of companion biomarkers to drugs to achieve the highest possible response rate with the minimal toxicity. The outcome of our methodology is an optimal drug catalog, the assignment of optimal biomarkers to each drug and a treatment decision protocol where a drug is used to treat a patient when the latter is positive for the drug companion biomarker. The application of the treatment decision protocol for every drug in the catalog results in optimal personalized combinatorial therapies for every patient.

An interest future direction is the investigation of the impact of drug interactions. We expect that the optimization approach will favor drugs that synergize with many other drugs in the catalog relative to those that do not interact or antagonize with other drugs in the catalog. At the end, the interplay between manifesting a high response rate in a group of patients and the degree of synergy (or absence of antagonism) with other drugs in the catalog will determine the suitability of a given drug for its use in personalized combinations. The challenge will be to estimate of the degree of synergy/antagonism between current anticancer drugs.

Our methodology is entirely based on estimated response rates given a marker. The latter can be estimated from clinical trails testing each anticancer drug as a single agent, where all patients enrolled are tested for a set of predefined biomarkers. Using this information we can estimate the overall response rate given a marker, for each of the markers considered. In second step, we should select a cohort of patients where the status of all these biomarkers has been determined. This cohort could be, in principle, the union of all cohorts where the drugs were tested as single agents. Using the mutation status of each gene and the estimated response rates given a marker we can estimate the response rate of each patient in an approximate manner. With those estimates at hand we can then apply the methodology introduced here and make a prediction for the optimal drug catalog, the assignment of optimal biomarkers to each drug and a treatment decision protocol where a drug is used to treat a patient when it is positive for the drug marker. Finally, the predicted personalized combinatorial therapy should be tested in a two arms clinical trial to determine how it performs compared to the standard of care.

The optimization scheme introduced here can be generalized in several directions. We can improve the response rate calculation including drug interactions, provided the direction and the magnitude of those interactions is given. Our approach is also suitable for the inclusion of genetic markers affecting drug metabolism [2]. These markers can be included in the optimization scheme as well, provided we specify a model for their impact on the response rate. Further generalizations are also needed to model toxicity. However, these generalizations will result in more complicated models with more parameters, many of which will be difficult to quantify. In the mean time, the simplifications introduced here allow us to implement the personalized combinatorial therapies approach in the clinical context, by routinely sequence a subset of genes on each patient enrolled in clinical trials.

## Methods

### Simulated annealing algorithm

**Figure 6:**
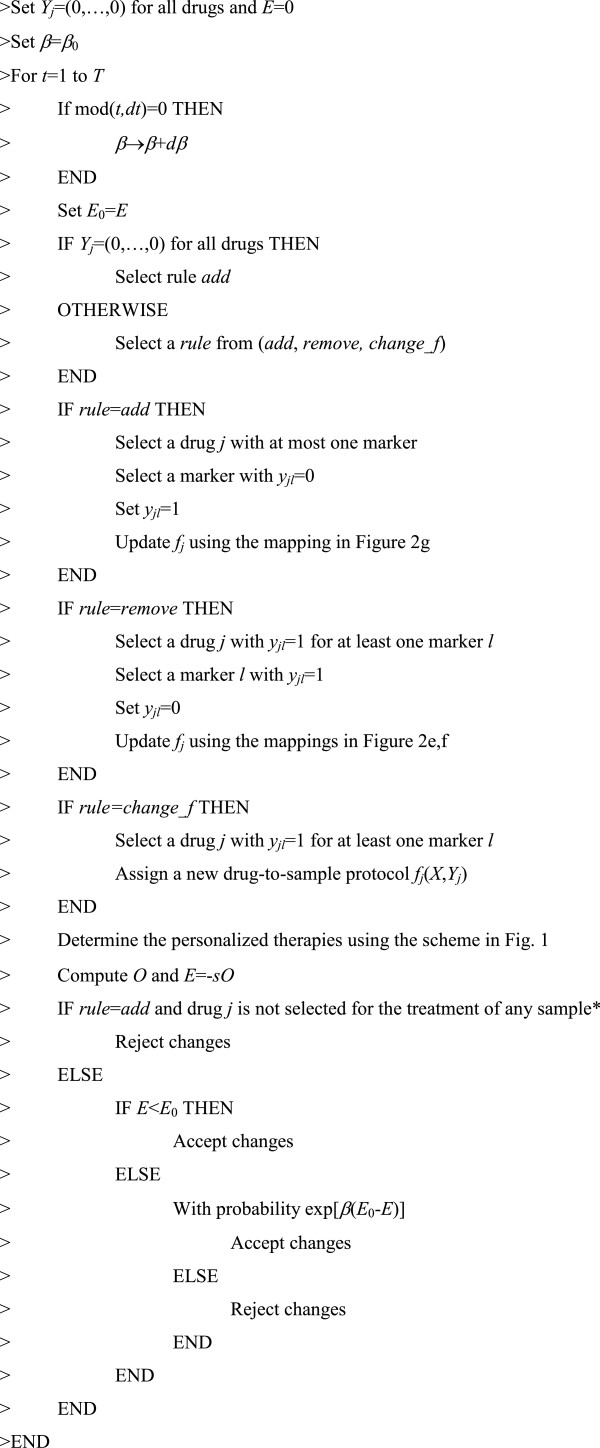
**Pseudocode for the simulated-annealing algorithm.** *This step is introduced to avoid the accumulation of markers in drugs that are not used for treatment.

The simulated-annealing algorithm aims to maximize the overall response rate, or equivalently to minimize *E*=−*sO*, where *s* is the number of samples. The algorithm starts from no markers assigned to drugs (*Y*_*j*_=(0,…,0) for all drugs) and explores random changes of the *Y*_*j *_and the drug-to-sample protocols *f*_*j*_(*X*,*Y*_*j*_). At each step of the algorithm, a drug *j* is selected and, for that drug, either a marker is added or removed or a new drug-to-sample protocol is selected. Changes are accepted when *E* decreases, and when *E* increases they are accepted with probability exp(β(*E*_0_-*E*)), where *E*_0 _and *E* are calculated before and after the change, respectively, and β is the “annealing” parameter. β is gradually increased such that, as the algorithm proceeds, changes increasing *E* are more likely to be rejected. The pseudocode for the simulated annealing algorithm implementation for our specific optimization problem is shown in Figure 6.

In our simulations we have chosen the parameters *T*=10,000*d*, *dt*=*d*, β_0_=0 and *d*β=0.01.

The simulated-annealing algorithm can get trapped in drug marker assignments that are suboptimal. To overcome this limitation we repeat the algorithm several times and report the solution with minimum *c*. We did not observe significant changes from a 100 to a 1,000 repetitions. The results discussed below are obtained for 1,000 repetitions.

### IC50 imputation

The missing logIC50 data was imputed using the weighted average over samples with available data

(7) $${\text{logIC}50}_{\mathrm{ij}}^{\text{Imputed}}=\frac{\sum_{k=1,k\neq i|{\text{logIC}50}_{\text{kj}}\neq \mathrm{NA}}^{s}{\text{logIC}50}_{\text{kj}}e^{-\alpha_{\text{sample}}d_{\text{ikj}}}}{\sum_{k=1,k\neq i|{\text{logIC}50}_{\text{kj}}\neq \mathrm{NA}}^{s}e^{-\alpha_{\text{sample}}d_{\text{ikj}}}}$$

where *NA* denotes missing value,

(8) $$d_{\text{ikj}}=\frac{\sum_{l=1|l\neq j|{\text{logIC}50}_{\text{il}}\neq \mathrm{NA}|{\text{logIC}50}_{\text{kl}}\neq \mathrm{NA},}^{d}{\left({\text{logIC}50}_{\text{il}}-{\text{logIC}50}_{\text{kl}}\right)}^{2}e^{-\alpha_{\text{drug}}d_{\text{jl}}}}{\sum_{l=1|l\neq j|{\text{logIC}50}_{\text{il}}\neq \mathrm{NA}|{\text{logIC}50}_{\text{kl}}\neq \mathrm{NA},}^{d}e^{-\alpha_{\text{drug}}d_{\text{jl}}}}$$

is a weighted distance between samples, and

(9) $$d_{\text{jl}}=\frac{\sum_{i=1|{\text{logIC}50}_{\text{ij}}\neq \mathrm{NA}|{\text{logIC}50}_{\mathrm{il}}\neq \mathrm{NA},}^{s}{\left({\text{logIC}50}_{\mathrm{ij}}-{\text{logIC}50}_{\text{il}}\right)}^{2}}{\sum_{i=1|{\text{logIC}50}_{\text{ij}}\neq \mathrm{NA}|{\text{logIC}50}_{\text{il}}\neq \mathrm{NA},}^{s}1}$$

is the Euclidean distance between drugs based on the available data. The exploration of the parameters (*α*_*sample*_, *α*_*drug*_) in the range (1–22,1-22) resulted in Pearson Correlation Coefficients (PCCs) between imputed and actual logIC50s, when available, in the range 0.83-0.89, with a maximum of 0.89 for (*α*_*sample*_ = 20, *α*_*drug*_ = 3).

## Competing interests

The authors declare they have no competing interests.

## Author contribution

AV conceived, executed and wrote this work.
